# The SEED and the Rapid Annotation of microbial genomes using Subsystems Technology (RAST)

**DOI:** 10.1093/nar/gkt1226

**Published:** 2013-11-29

**Authors:** Ross Overbeek, Robert Olson, Gordon D. Pusch, Gary J. Olsen, James J. Davis, Terry Disz, Robert A. Edwards, Svetlana Gerdes, Bruce Parrello, Maulik Shukla, Veronika Vonstein, Alice R. Wattam, Fangfang Xia, Rick Stevens

**Affiliations:** ^1^Fellowship for Interpretation of Genomes, Burr Ridge, IL 60527, USA, ^2^Mathematics and Computer Science Division, Argonne National Laboratory, Argonne, IL 60439, USA, ^3^Computation Institute, University of Chicago, Chicago, IL 60637, USA, ^4^Department of Microbiology, University of Illinois at Urbana-Champaign, Urbana, IL 61801, USA, ^5^Department of Computer Science, San Diego State University, San Diego, CA 92182, USA, ^6^Virginia Bioinformatics Institute, Virginia Tech, Blacksburg, VA 24060, USA, ^7^Computing, Environment and Life Sciences, Argonne National Laboratory, Argonne, IL 60439, USA and ^8^Department of Computer Science, University of Chicago, Chicago, IL 60637, USA

## Abstract

In 2004, the SEED (http://pubseed.theseed.org/) was created to provide consistent and accurate genome annotations across thousands of genomes and as a platform for discovering and developing *de novo* annotations. The SEED is a constantly updated integration of genomic data with a genome database, web front end, API and server scripts. It is used by many scientists for predicting gene functions and discovering new pathways. In addition to being a powerful database for bioinformatics research, the SEED also houses subsystems (collections of functionally related protein families) and their derived FIGfams (protein families), which represent the core of the RAST annotation engine (http://rast.nmpdr.org/). When a new genome is submitted to RAST, genes are called and their annotations are made by comparison to the FIGfam collection. If the genome is made public, it is then housed within the SEED and its proteins populate the FIGfam collection. This annotation cycle has proven to be a robust and scalable solution to the problem of annotating the exponentially increasing number of genomes. To date, >12 000 users worldwide have annotated >60 000 distinct genomes using RAST. Here we describe the interconnectedness of the SEED database and RAST, the RAST annotation pipeline and updates to both resources.

## INTRODUCTION

Starting in the mid-1990s, entire bacterial and archaeal genomes were beginning to be sequenced. These early sequencing projects were large undertakings, fraught with technical challenges and requiring thousands of man-hours to complete. Major obstacles resulted from limitations in sequencing technology and the onerous task of determining the functions of each gene. Early on, genome annotation was largely a by-hand effort, and it could take an individual researcher several months to annotate a single megabase of DNA (1,2). Depending on the organism, the end result was a somewhat dissatisfying reflection of the current knowledge of the field. For instance, at the time only 62% of the genes in *Escherichia coli* K-12 could be assigned a functional role (3). In organisms that were not as well studied this number was far worse; for instance, only 38% for the archaeon *Methanocaldococcus jannaschii* (4). In the past 16 years these numbers have improved with >90% of the genes in *E. coli* K-12 and ∼70% of the genes in *M. jannaschii* having a known functional role (5–7). These gains have been achieved through direct research on these organisms and the integration of data from research on other organisms.

From its inception in 2004, the goal of the SEED project has been to integrate annotations from a wide variety of sources and to use them to improve our knowledge about microbial genomes (5). Many scientists are experts in a circumscribed area of physiology or metabolism. By capturing information from individual scientists in annotated subsystems, we leverage their expertise in the annotation and analysis of all microbial genomes, not just the few model systems that are well studied. Thus, each genome covers the expertise of a wide range of biologists that would not have otherwise been used if individual genomes had been annotated one-by-one. The initial investment in manual curation by skilled biologists building subsystems that include all available genomes has now formed the basis of many thousands of automated annotations at high levels of accuracy. We believe that automated annotation systems, like the one used by the SEED, will ultimately reach the point where they can match the performance of the most skilled human annotators; and they will reach this point *via* incremental improvements where limited amounts of manual annotation play a central role.

## THE SEED

The SEED continually integrates different types of genomic data from a variety of sources. These include public genomes annotated by RAST (8), expert user annotations, metabolic modeling data (9,10), expression data, literature references verifying annotations (11) and links to data from other popular resources including Swiss-Prot (12), GenBank (13), IMG (14), KEGG (15), CDD (16) and so forth. These data are made accessible primarily in two ways: through web access (5) and high-performance computing servers that are accessible programmatically *via* an API and server scripts (17) (tutorials are available at http://www.theseed.org/).

### The SEED Web site (SEED viewer)

**Figure 1.**
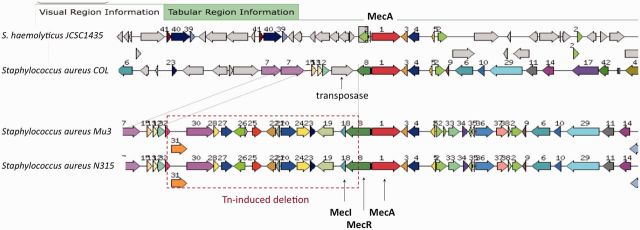
The ‘Compare Regions’ tool in the SEED. The Staphylococcal SCCmec element is shown as an example. Re-arrangements within Staphylococcal SCCmec element lead to constitutive expression of resistance determinant MecA due to (partial) deletion of repressor MecI and/or sensor-transducer MecR. Homologous genes are presented as arrows with matching colors and numbers. Genes not conserved within the displayed region are gray. The graphic is centered on the focus gene (red, #1): Methicillin resistance determinant MecA; green, #8: Methicillin resistance regulatory sensor-transducer MecR1; blue, #18: Methicillin resistance repressor MecI; green, #2: transposase for IS431.

The SEED Web site presents a rich environment for genome annotation and comparison. Inspired by the Google search page, the SEED start page has also a single window, which allows the user to search for a genome of interest, a gene, a protein, a feature or a functional role. The same page provides dropdown menus for other entries into the SEED Viewer environment. Registration to the SEED is only required for users that would like to make changes to the database. For each protein in a genome, the SEED Web site offers a protein page that contains direct links to the NCBI CDD database (16), the KEGG Enzyme database (15) and PubMed ID links to articles describing the functional role of a given gene product (11) (15 565 links). Perhaps the most popular tool on the SEED Web site is the ‘Compare Regions View’, which is an integral part of each protein page. This tool allows users to compare the genomic neighborhood of a given gene across genomes. The user has the ability to set the number of genomes that the gene of interest is compared with, the similarity threshold for inclusion in the comparison, the coloring of genes based on similarity and the size of the region being displayed. This tool provides a powerful means for finding and correcting gene calls and for predicting new functions based on conserved genomic context (Figure 1). Many protein pages now have links to pre-computed alignments and trees. For some of the SEED organisms the protein page also has links to expression data that has been pre-processed to present ‘Atomic Regulons’, sets of co-expressed genes. Information of this kind is invaluable when disambiguating the products of paralogous genes (18).

**Figure 2.**
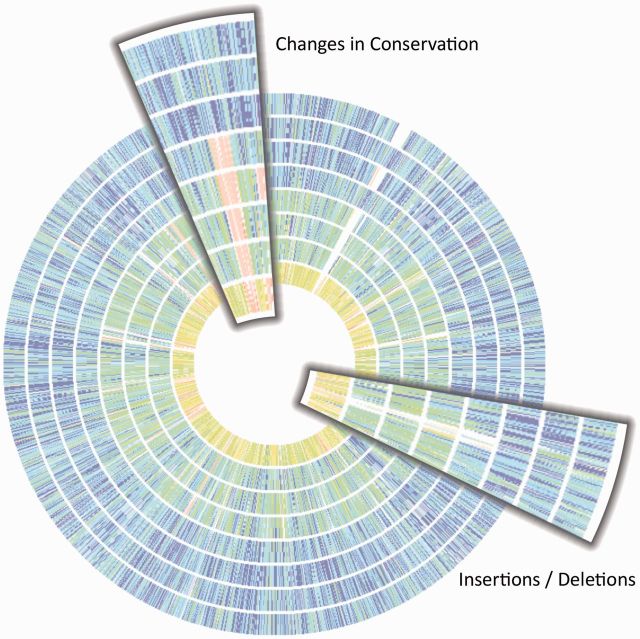
Circle plot showing the comparison of eight *Brucella* genomes relative to a user-defined reference genome. The zoomed regions highlight insertions/deletions (colored versus white) and changes in conservation relative to the reference genome (going from blue representing the highest protein sequence similarity to red representing the lowest).

The SEED and RAST Web sites support a multitude of comparative genomics tools. For example, as shown in Figure 2, users can readily identify insertions and deletions in up to nine target genomes compared with one reference genome using the ‘Sequence Based Comparison Tool’. The tool colors each gene based on protein similarity using BLAST (19), and each gene is marked as being unique, a unidirectional best hit or a bidirectional best hit in comparison to the reference genome. The output also includes a whole-genome schematic colored by BLAST similarity and BLAST dot-plots between compared organisms. The resulting data table can also be downloaded for further analysis. Like the ‘Sequence Based Comparison Tool’, the ‘Function Based Comparison Tool’ compares two genomes to assess similarities and differences in the presence of functional roles that have been linked to subsystems. This enables the user to view unique functions found in either genome. Results of this analysis can also be downloaded for further study.

The SEED Web site also allows users to browse the current collection of subsystems, which are proteins grouped by a relationship in function (5). For instance the subsystem ‘tRNA aminoacylation Phe’ includes the functional roles, ‘Phenylalanyl-tRNA synthetase alpha chain (EC 6.1.1.20)’ and ‘Phenylalanyl-tRNA synthetase beta chain (EC 6.1.1.20)’. The subsystem spreadsheet is populated with all genomes that have those functional roles and provides links to the relevant protein pages. The subsystem info tab provides an expert annotator's notes on the creation of the subsystem. Although they are not comprehensive, the SEED subsystems are a particularly useful way to quickly determine the proteins that are involved in a related function and to determine known variations in functionality between organisms. Experts in areas of microbial biochemistry and physiology are encouraged to annotate genes on the pubic version of the SEED (http://pubseed.theseed.org), so that their knowledge can be propagated to the scientific community.

### Programmatic access to SEED data

A network-based API allows programmatic access to all of the data that exist within the SEED (17). A comprehensive set of tutorials for accessing data and the software necessary to interact with the SEED servers can be found here (http://www.theseed.org/servers/). SEED data can be accessed *via* four different servers: the Sapling server contains genomic data, the ANNO server supports capabilities relating to annotation, the RAST server enables batch submission to RAST and the Model server provides access to metabolic modeling data underlying the Model SEED (9).

As most of the API access routines are used repeatedly and writing new code can be labor intensive, the SEED also offers a large repository of >150 server scripts (http://pubseed.theseed.org/sapling/server.cgi?pod=ServerScripts). Each server script is a small program that accesses the SEED servers from the command line. These server scripts perform a multitude of common tasks. For example, ‘svr_all_genomes’ will return the scientific name and genome identifier for every genome in SEED, and ‘svr_function_of’ returns the functional role for a given protein identifier. The server scripts can be piped together to create a powerful suite of bioinformatics tools, yet require little programming knowledge to use. The SEED server scripts are distributed as part of the myRAST installation (described later in text).

### SEED-supported resources

The use of a standard vocabulary and continual improvement of genome annotations coupled with a robust database structure has made the SEED project an attractive venue for several productive collaborations (Table 1). The SEED currently offers data supporting NMPDR, the **N**ational **M**icrobial **P**athogen **D**ata **R**esource (unfunded, Web site operational) (20); PATRIC, the **P**athosystems **R**esource **I**ntegration **C**enter; the all-bacterial BRC (**B**ioinformatics **R**esource **C**enter) (http://www.patricbrc.org) (21); PhAnToMe, **Ph**age **An**notation **To**ols and **Me**thods (http://www.phantome.org) (unfunded, Web site operational); Model SEED (9) and the U.S. Department of Energy KBase project (in progress).

**Table 1.** Online resources supported by SEED technology

Resource	Input	Usage	Description	URL
PubSEED	Genome, gene, protein, functional role, pathway (text search and sequence search)	• Browse SEED and explore SEED-based knowledge about the feature of interest • Find contextual clues based on gene co-localization, fusion events, phylogenetic profiling • Compare genomes (sequence based or function based) • Explore subsystems • Browse pre-computed alignments and trees for protein of interest • Register as user and get annotation rights (add/change annotations, build subsystems, add literature and so forth.)	Genome database and collection of tools designed for high-quality genome annotation and comparative genome analysis for research applications; genome context analysis tools use gene co-localization, fusion events, phyletic (occurrence) profiling; the only major database editable and expandable by a user (on registration); intended for experimental biologists, does not require programming skills	http://pubseed.theseed.org/
RAST	DNA sequence (genome, phage, plasmid)	• Download RAST-annotated genome (gene calls, protein functions, subsystems) and use your own tools • Browse RAST-annotated genome in SEED Viewer (compare with public genomes or other genomes that you have submitted to RAST) • Curate your RAST-annotated genome in SEED Viewer (change annotations, add/delete gene calls) • Allow collaborators pre-publication access to your RAST-annotated genome • Request automatic metabolic model when submitting your genome to RAST	Automatic server for rapid and accurate annotation of prokaryotic, phage or plasmid genomes using SEED technology	http://rast.nmpdr.org/
myRAST	DNA sequence (prokaryotic genome or metagenomic data)	• Download and install locally a myRAST distribution package • Perform automated and manual annotation of private genome or metagenome on your laptop • Use pre-programmed scripts (>150 available) • Extract various types of data from SEED or run numerous computational tasks remotely	Standalone application for a user's computer capable of performing computationally expensive operations (e.g. annotation of genomes or collections of metagenomic sequences) using SEED web service technology	http://blog.theseed.org/servers/introduction.html
Server scripts	Research questions	• Download and install locally a small Client Package (Perl or Java) that defines network-based SEED API • Use pre-programmed scripts (>150 available) or pipe them together • Extract various types of data from SEED or run numerous computational tasks remotely	High-performance network-based servers that provide programmatic access to all data types in SEED: genomes, annotations and metabolic models	http://pubseed.theseed.org/sapling/server.cgi?pod=ServerScripts
ModelSEED	RAST-annotated genome	• Generate draft genome-scale metabolic model starting from a RAST-annotated prokaryotic genome sequence • Compare two or more models for the same organism or for diferent species • Predict culture conditions for an organism • Predict essential genes	Public resource for the generation, optimization, exploration, comparison and analysis of genome-scale metabolic models	http://seed-viewer.theseed.org/models
PATRIC	bacterial taxon, genome, gene, pathway, transcriptomic data, (text search, sequence search, metadata-based filtering and browsing)	• View and analyze RAST-annotated genomes, compare annotations from different sources • Compare protein families and pathways across hundreds of genomes using interactive analysis and visualization tools • View, analyze and compare public and private transcriptomic data sets • Use metadata-based filtering and smart searches to find data of interest • Analyze protein–protein interactions and disease-associated data • Work in private workspace and save default data sets	The all bacterial bioinformatics resource center (BRC) that provides integrated data and analysis tools, intended as a resouce for experimental biologists, tries to meet the needs of both bioinformaticians and the computationally naïve user	http://www.patricbrc.org
PhAnToMe	phage or prophage	• Browse phage database • Identify prophages in microbial genomes • Compare phages and prophages in SEED Viever • Explore phage subsystems	Phage and prophage annotation database with a visual programming interface	http://www.phantome.org/

## RAST

RAST, **R**apid **A**nnotations using **S**ubsystems **T**echnology (8), is an automatic annotation server for microbial genomes, built upon the framework provided by the SEED system. A new user must register for the service, which involves giving us contact information and acquiring a password. By registering users, we can create a framework in which users have access to only those genomes that they have submitted. It allows us also to contact the user once the automatic annotation has finished or in case user intervention is required. RAST is designed to consistently produce annotations comparable in quality to those produced by the best human annotators and to extend those annotations to as many protein-encoding genes in as many genomes as possible. Continuous addition of new subsystems that cover previously un-annotated regions of the genomes, and continuous quality control of existing subsystems are central to improved annotations in the SEED and their propagation *via* FIGfams into RAST (5,8,10,22,23). RAST-annotated public genomes are then introduced into the SEED and included in the SEED curation. The SEED => FIGfam => RAST => SEED cycle is at the heart of SEED-based annotations.

**Figure 3.**
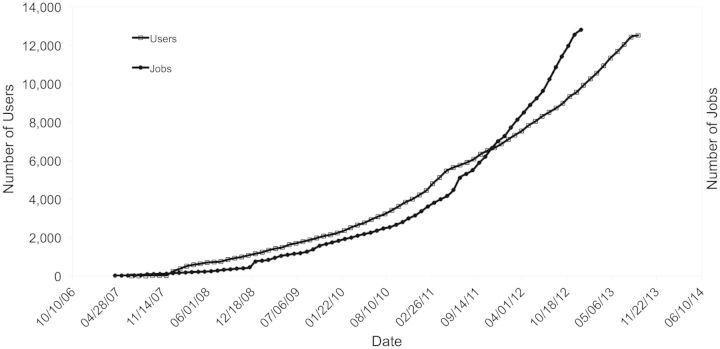
Number of users (open squares) and number of jobs (closed circles) in the RAST system. As of September 2013, there were over 100 000 jobs processed by RAST and >12 000 active users of the system.

RAST was introduced in 2007, and concomitant with the plummeting cost of DNA sequencing, we have seen the number of genomes annotated by RAST increase by >2 orders of magnitude, from 350 genomes in the initial release to >60 000 distinct genomes (>100 000 jobs submitted) annotated to date (Table 2). Although the number of jobs continues to grow (Figure 3), the average time to compute a job has decreased slightly over the years (data not shown) as both faster computers are deployed to our infrastructure and improvements to our algorithms are incorporated in our code base. Currently, the RAST server is used routinely to annotate 200–300 prokaryotic genomes daily (up to 700 at peak loads), of which over two-thirds are unique and >1 Mb long. In the next 5 years, we anticipate annotating hundreds of thousands of microbial genomes.

**Table 2.** Major milestones and improvements in the RAST system over the past 5 years

Categories	2008	2013
Users	120	12 000
Jobs	1200	100 000
Distinct genomes	350	60 000
Number of FIGfams	100 000	185 000
Number of PEGs in FIGfams	1.1 million	16 million
Throughput	50–100 genomes/day	500–1000 genomes/day
Maximum throughput	300 genomes/day	1000 genomes/day
Number of subsystems	700	1600
Number of literature references attached to features	19 562	1 349 874
Data types accepted	Complete genomes	Phages, plasmids, draft genomes, complete genomes
Formats accepted	FASTA	FASTA, GenBank
Submissions	Single, web-based submissions only	Web submissions and batch submissions
ORF calling	Glimmer2	Gimmer3, RAST, user provided ORF calls

All of the nearly 12 000 bacterial genomes available from PATRIC have been consistently annotated using RAST. PATRIC provides researchers with a resource that stores and integrates a variety of data types (genomics, transcriptomic, protein–protein interactions, 3D protein structures and sequence typing data) with their associated metadata. Data are summarized at the level of the individual genome and across taxonomic levels (21). PATRIC also allows researchers to compare RAST annotations with those from other sources, most notably annotations from GenBank/RefSeq.

**Figure 4.**
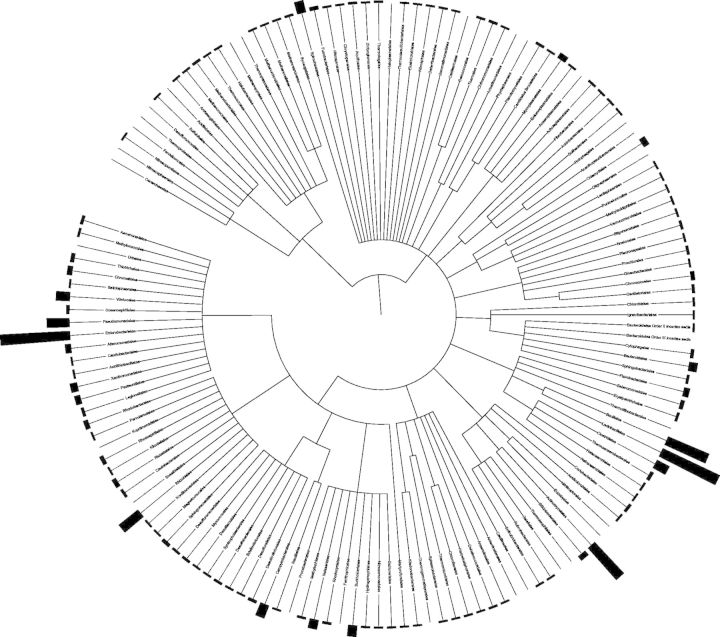
Genomes processed by RAST displayed over a taxonomic tree. In all, 12 289 RAST annotated public genomes for PATRIC available on the PubSEED were compared at the order level using the NCBI taxonomy (25). Black bars show the number of sequenced representatives per order. White bars show those orders with no sequenced representatives. The tree was created using the Interactive Tree of Life (http://itol.embl.de/) and is unrooted.

Figure 4 shows the genomes annotated by RAST for PATRIC displayed on a taxonomy-based tree for the orders in the bacteria and archaea (24). All of those genomes (unlike other RAST annotated genomes) are public. They can be used to visualize the great diversity of genomes that have been annotated by RAST.

### The RAST pipeline

The RAST pipeline implements the following steps to annotate a prokaryotic genome:

1. Identify the selenoproteins and pyrrolysoproteins. These special case genes are sought using custom algorithms. There is a growing set of such special cases where domain-specific knowledge is required to recognize the genes and most alignment programs such as BLAST are not sensitive enough to discriminate between the special-case gens and the similar but non-special-case genes.
2. Generate an estimate of the 30 closest phylogenetic neighbors in the SEED by comparing *ab initio* GLIMMER3 gene-candidates with a set of universal proteins plus up to 200 ‘unduplicated’ proteins (26). These gene candidates are only used to identify the phylogenetic neighborhood and to help ‘bootstrap’ iterative retraining of GILMMER3 and are not retained in the final annotation.
3. Identify the tRNA and rRNA genes using ‘search_for_rnas’ (Niels Larsen, unpublished, available from the author on request), which uses tRNAscan-SE to find tRNAs (27) and BLASTN (19) against a set of RNA databases followed by endpoint adjustment to find rRNAs.
4. Test all of the gene candidates from step 2 to identify those that are similar to proteins in subsystems using signature amino-acid *k-*mers (sets of eight sequential amino acids). The *k-*mers allow us to rapidly scan the gene candidates against all known proteins, as we have described for metagenomes elsewhere (28). Candidates having *k-*mer evidence for a subsystem-based function are ‘promoted’ to the status of ‘protein-encoding gene’ (PEG), and assigned ≥1 functional roles based on that *k-*mer evidence.
5. Iteratively retrain GLIMMER3 on the set of gene candidates validated by *k*-mers in step 4. Steps 4 and 5 are repeated until no new gene candidates are found that are similar to those in subsystems. Gene candidates are only retained if they match a gene in a subsystem and do not significantly overlap a gene that was called previously. In practice, convergence is usually achieved after three iterations and ‘overtraining’ is not observed.
6. Any remaining gene candidates that do not significantly overlap an existing gene call are included if they are similar to any protein in the 30 closest neighbors using BLASTP (19).
7. Any remaining gene candidates that do not significantly overlap an existing gene call are included.
8. Gene fragments that may contain frameshifts due to low-quality sequencing are detected by comparing with the template genes in the 30 nearest neighbors. If requested by the user, these gene fragments are joined to a single gene, and detailed statements of what was inferred and why are recorded.
9. Any DNA stretches longer than 1500 bp that do not contain a gene are ‘backfilled’ with gene candidates by comparing them with the proteins from the 30 nearest neighbors using BLASTX (19).
10. Functions are assigned to products of genes without *k-*mer-based assignments by using BLASTP similarities.
11. If a gene candidate has not been assigned a subsystem-based functional role, and it has flanking genes with subsystem-based functional roles, then it is compared with the nearest neighbors from step 2. If all three genes are bidirectional best hits (BBHs) to the corresponding set of three genes in a neighboring genome, then the current assignment is replaced by the subsystem-based functional role from the neighboring genome.
12. Missed genes are identified by examining remaining gaps flanked by genes that are BBHs to genes that are in subsystems in a neighboring genome.
13. Gene candidates that do not have subsystem or BLAST support and are embedded within another gene, significantly overlap a gene or are extremely short (<90 nt) are removed.
14. Subsystem analyses and initial metabolic reconstructions are performed. The subsystems analysis calculates which subsystems are reflected in the genome, and for each subsystem estimates the most likely variant. The metabolic reconstruction connects the annotations to the metabolic model in preparation for flux balance analyses in the Model SEED (9).
15. Pairs of close bidirectional best hits (PCBBHs) are computed against genomes in the PubSEED. These support estimates of functional coupling based on conserved contiguity (29,30).
16. Genome data are exported in GenBank, EMBL, GFF3, GTF, Excel and tab-delimited formats.

Due to its popularity, there have been many attempts to use RAST to annotate chunks of DNA that were not contigs in prokaryotic genomes. Because of the iterative approach of the annotation algorithm and the reliance on closely related genomes, RAST is not able to annotate mixed sequences (e.g. mixed culture genomes, metagenomes). However, we have adapted the RAST pipeline to annotate phage and plasmid genomes, which often have close homologs. The phage/plasmid pipeline (invoked automatically for submissions of <100 kb in all contigs) involves finding the RNAs and close neighbors using the pipeline described earlier in text, but substituting MGA (31) for GLIMMER3 in the initial gene calling step. Step 5 of the pipeline, the iterative gene calling, is only run once, and all candidate genes are accepted. All subsystems are used to annotate the phage genes, but the ∼50 phage-specific subsystems introduced by the PhAnToMe project (http://www.phantome.org/) enhance the quality of phage-specific genome annotations. The pipeline then skips forward to Step 8, identifying and repairing frame shifts, and the rest of the pipeline continues as described.

### Manual improvements to RAST-annotated genomes

The RAST user interface (derived from the SEED interface) allows registered users to make manual changes to their genomes before retrieving them. The user can elect to delete or add gene calls, adjust start positions for genes, change functional role annotations and re-compute the subsystems asserted. Tutorials on manual annotation are available from the RAST entry page.

### myRAST

We have implemented several high-performance web services for computation against SEED data (17). These SEED web services may also be accessed *via* a standalone application called myRAST, a demonstration project built using SEED web service technology. myRAST supports automated and manual annotation of both genomic data and collections of metagenomic (DNA) data. Genomic data are annotated using the SEED servers to identify protein-encoding genes and RNA genes similar to the RAST pipeline described earlier in text, and to annotate the protein-encoding genes using the SEED *k-*mer-based annotation algorithm (28). The annotated genomes are installed into a local (to the user's computer) relational database using the SEED ERDB technology. myRAST is freely available for download from the web at (http://blog.theseed.org/servers/installation/distribution-of-the-seed-server-packages.html). An article describing myRAST in detail is in preparation.

The myRAST application also computes an estimate of the genomes most closely related to the user's genome, and then computes a set of fairly conservative correspondences between the user's genome and each genome in this set. These data are used to drive the myRAST compare regions viewer, which is similar to the compare regions viewers in the SEED and in RAST.

myRAST may also be used to load and visualize the SNP analysis available in the SEED toolkit. Here, a set of user genomes is analyzed in comparison with a single reference genome. This analysis generates gene calls and annotations as propagated from the reference genome, as well as a set of SNPs occurring in both the genes and the intergenic regions. For each SNP the user may view the corresponding DNA or protein alignments.

## FUTURE DEVELOPMENTS

Due to increasing demand, RAST will soon support annotating organisms from the same species using a reference genome specified by the user. When specified, an attempt will be made to inherit all annotations from the reference genome and also propagate gene names. Because gene names are used inconsistently across species, neither the SEED nor RAST has ever attempted to propagate them (32). For example, the gene *sir*A of *Salmonella* is also known as *uvr*Y in *E. coli* or *gac*A in *Pseudomonas*. Instead, the SEED and RAST attempt to consistently propagate subsystem-based functional roles.

Performance in RAST is a constant issue, especially in the face of exponentially increased use. We have recently installed changes that allow us to process >700 jobs per day. Although we expect to improve performance further, our efforts are now largely directed at achieving improved accuracy (10,23). We are also planning to redesign the user interface for the SEED and RAST to accommodate the wealth of genomes. The community is constantly producing tools that recognize, and often characterize, specific classes of genome features. We are planning to add several more of these new specialized tools to our pipeline, such as the recognition of BOX elements in Streptococci (33) and the identification of CRISPRs (34) and so forth.

We intend to institute a ‘Publish to PATRIC’ button that will allow users to immediately share their genomes publicly through the PATRIC portal. The PATRIC identifier can then be used in publications to direct others to the annotated genome product. Genomes that have been exported to PATRIC can then use the wide suite of tools that PATRIC has to offer to explore and compare annotated genomes, and to compare annotations from a variety of sources.

## FUNDING

United States National Institute of Allergy and Infectious Diseases, National Institutes of Health, Department of Health and Human Service [HHSN272200900040C], the National Science Foundation Grant [DBI-0850546], as well as the Office of Science, Office of Biological and Environmental Research, of the United States Department of Energy [DE-AC02-06CH11357], as part of the DOE Systems Biology Knowledgebase. United States National Science Foundation Grant [DBI-0850356] (to R. A. E.) from the NSF Division of Biological Infrastructure (the PhAnToMe project). Funding for open access charge: National Institute of Allergy and Infectious Diseases.

*Conflict of interest statement*. None declared.
